# Complex aortic valve repair in congenital patients: clinical feedback

**DOI:** 10.3389/fped.2024.1466311

**Published:** 2024-11-19

**Authors:** Marie-Anne Barbier, Nicolae Cristian Bulescu, Olivier Metton, Lea Bou Karam, Caroline Martin Bonnet, Anne Moulin Zinsch, Marc Lilot, Roland Henaine

**Affiliations:** ^1^ Congenital Cardiac Surgery, Louis Pradel Hospital, Lyon, France; ^2^ Pediatric and Congenital Cardiology Department, Hopital Louis Pradel, Lyon, France; ^3^ Pediatric Cardiac, Thoracic and Vascular Anesthesia and Intensive Care Unit, Louis Pradel Hospital, Lyon, France

**Keywords:** aortic valve repair, root surgery, complex aortic valve disease, congenital heart disease, pediatric cardiac surgery

## Abstract

**Introduction:**

In the congenital population, particularly in young adults, the best strategy for aortic valve surgery has not been clearly established. This study investigates the mortality, perioperative morbidity and echocardiographic evolution of complex aortic valve repair techniques.

**Material and methods:**

We performed a retrospective monocentric descriptive study of patients operated at the Louis Pradel Hospital (Lyon) from 2017 to 2023. We included patients operated for complex aortic valve repair by the congenital heart surgery team, excluding simple commissurotomies. The primary endpoint was postoperative survival. The secondary endpoints were freedom from surgical reintervention and echocardiographic evolution of aortic regurgitation, aortic stenosis and annular diameter. The analysis was performed using Kaplan Meier methods.

**Results:**

Twenty-eight patients were included. The mean age was 11.1 years (range 1–35 years) and the mean weight was 37.5 kg (range 8.2–79 kg). The postoperative survival rate was 96.4% at discharge (27/28 patients). The mean follow-up interval was 35 months (range 14–79 months). At the end of the follow-up, the freedom from reoperation was 85.7%. Four patients underwent reoperation for worsening aortic valve and/or ventricular function (1 heart transplantation, 2 Ross procedures, 1 aortic valve replacement). Results on secondary endpoints showed a trend towards improvement in annular diameter postoperatively between pre- and postoperative echocardiography for each patient, with no statistically significant difference for aortic insufficiency, but for aortic stenosis (*p* = 0.02).

**Conclusion:**

This study shows an excellent survival rate, and a similar risk of reintervention compared with literature data. All the data described above argue in favor of an aortic valve repair surgery as a first line procedure in case of congenital heart disease.

## Introduction

The choice of valve repair techniques in the management of aortic valve disease is still poorly guided, especially in young patients or in those with high risk of complications (1). These include specific procedures done at the level of the aortic valve cusps (bicuspidisation or patch repair as the most frequently used techniques), external annuloplasties and procedures to treat aortic root aneurysms. The numerous advantages of aortic valve repair have been described, such as no necessity for a lifelong anticoagulation therapy, nor an accelerated degeneration or calcification (as in case of biological prostheses or homografts, respectively). The secondary dilatation of the root appears to be less frequent than for the Ross procedure. The risk of infection is also reduced (2).

The choice of technique is made by a heart team, and it depends on the mechanism of the leak (3) and the aortic diameters, according to pre-established diagrams (4). In the case of annular dilatation with aortic insufficiency, it has been shown that stabilization of the aortic annulus by external annuloplasty takes an important part in the durability of valve repair procedures. The CAVIAAR study showed excellent results in terms of safety and durability at 10 years, when combined with well-managed valve repair (5).

The aim of this study was to investigate the mortality of complex aortic valve repair techniques in short and medium term. Secondary endpoints were perioperative morbidity, and evolution of echocardiographic criteria over the time.

## Material and methods

### Patients

The study consisted in a retrospective analysis of the patien's clinical records followed for congenital heart disease who received complex aortic valve repair by one of the surgeons of the Congenital Cardiac Surgery team, between May 01, 2017 and March 01, 2023 at the Louis Pradel Hospital using Crystal Net and Easily bloc software. Patients were analyzed on a per-protocol basis, taking into account only cases where a successful valve repair was obtained at the end of the procedure. Cases where a rescue valve replacement was required have not been included in this study.

**Figure 1:**
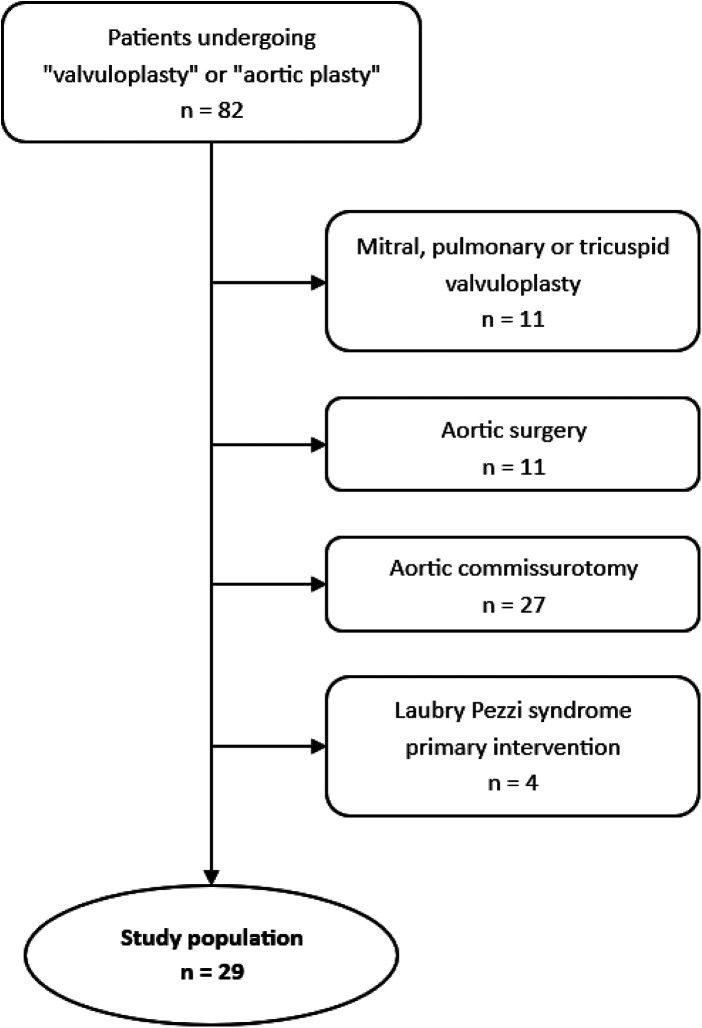
Flow chart.

Patients who underwent a valvuloplasty procedure on the mitral valve, pulmonary valve or tricuspid valve, and aortic surgery without a procedure on the aortic valve were excluded. Patients who underwent a simple valvuloplasty such as commissurotomy, or aortic valve surgery for Laubry-Pezzi syndromes, were also excluded. As a result, a total of 28 patients underwent an aortic valve surgery with a procedure considered as complex valvuloplasty (Figure 1).

The data collected from each patient's file was used to compile clinical and paraclinical information.

In total, several types of data were collected: demographic data (gender, sex, age, weight, height, etiology of valvular disease, indication for surgery, previous or subsequent surgeries), intraoperative data [duration of bypass surgery and clamping, procedure performed, need for reclamping, cardiac rhythm at cardio pulmonary bypass (CPB) exit], data concerning the stay in intensive care (duration of mechanical ventilation, weaning from amines, postoperative complications such as the need for dialysis or circulatory assistance) and echocardiographic data: aortic diameters, aortic insufficiency (AI) grade, aortic gradient, valve morphology, pulmonary arterial hypertension (PAH), mitral gradient, ventricular diameters, Left vlntricular ejection fraction (LVEF), fractional shortening (FS).

For all our patients, 4 echo scans were performed: one preoperatively, one upon discharge from hospital, one performed within the year following hospitalization (average 9 months), and the last echocardiography available in our databases (average 29 months).

Indications for intervention are detailed in the Results section.

### Surgical technique

All surgeries are performed under CPB, with cannulation of the ascending aorta and two caval veins, venting of the right superior pulmonary vein, aortic clamping, transverse incision of the ascending aorta extended to the non-coronary sinus, crystalloid cardioplegia, then execution of the specific procedure on the aortic valve.

In this cohort, the following procedures were performed: cusp plication, triangular cusp resection, pericardial patch on cusps, commissure suspension, bicuspidization, direct suture of defect (6–8). Similarly, several aortic root surgical techniques have been described, mainly the Yacoub procedure, David procedure (9) or annuloplasties if the surgery remains conservative (8, 10).

### Statistical analysis

Quantitative variables are presented as the mean and standard deviation, whereas qualitative variables are presented as the number and percentage.

ANOVA on repeated measures (library lme4) was performed to compare the values of quantitative variables at different measurement times. Survival curves using Kaplan-Meier methods will be produced for postoperative survival (90-day cut-off) and freedom from re-intervention (event = death or re-intervention). Fisher test were performed to compared evolution of AI during follow-up between patients who received patch repair vs. those without patch repair.

## Results

Indications for intervention were, for aortic stenosis, a mean gradient greater than 40 mmHg, and for aortic insufficiency, an estimated grade 3–4 leak on preoperative trans thoracic echocardiography associated with significant clinical symptoms. Valves had to be considered morphologically repairable on preoperative trans thoracic echography, assessed by a combined medical and surgical team.

Indications for a replacement of the ascending aorta in adults are: a diameter greater than 45 mm in patients with Marfan syndrome, 50 mm for patients with a bicuspid valve and risk factors, and 55 mm for patients without connective tissue disease (11). The maximum aortic diameter for intervention in children has not yet been clearly established, but it would seem that a maximum diameter of 200% or 160% of normal aortic diameter (for age and BSA) is appropriate if there is an associated valvulopathy or if the aneurysm is symptomatic (12). We have used the same thresholds for our clinical decision making.

### Population characteristics

Baseline characteristics are reported in Table 1, echocardiographic parameters are reported in Table 4. The population is mainly made of older children and teenagers (8–18 years old) (17 patients, 60.7%). Most of the patients have a congenital etiology of their aortic valve disease (20 patients, 71.4%). Most patients were operated for aortic regurgitation (18 patients, 64.3%). Thirteen patients (46.4%) had undergone prior cardiac surgery, of which 5 patients (17.9%) had undergone specific aortic valve surgery. Pre-operative transthoracic echocardiography revealed a majority of patients with aortic insufficiency of grade 2 (17.9%) or 3 (46.4%). For patients with AS, the mean maximal gradient over the aortic valve was measured at 61.8 mmHg. The median diameter of the aorta was 20.5 mm at the annulus. The morphological profile of the aortic valves shows a similar number of tricuspid (39.3%) and bicuspid (46.4%) valves.

**Table 1:** Baseline characteristics.

Perioperative patient data	Patients, *n* = 28
Age, years	11.1 [1–35] (7.7)
Children <8 Y	8 (28.6%)
Older children and teenagers: 8–18 Y	17 (60.7%)
Adults >18 Y	3 (10.7%)
Male sex	18 (64.3%)
Weight, kg	37.5 [8.2–79] (22.7)
Height, cm	134.7 [74–180] (34.7)
Diagnosis	
Aortic regugitation	18 (64.3%)
Aortic stenosis	7 (25%)
Mixed aortic valve disease	3 (10.7%)
Etiology	
Congenital	20 (71.4%)
Infective endocarditis	1 (3.6%)
Genetic syndrome (Turner, Down syndrome, Marfan, Wiliams Beuren, Loeys Dietz)	7 (25%)
History of surgical aortic valvuloplasty	5 (17.9%)
History of prior cardiac surgery	13 (46.4%)
Emergency surgery	4 (14.3%)
Aortic valve anatomy	
Unicuspid	4 (14.3%)
Bicuspid	13 (46.4%)
Tricuspid	11 (39.3%)

Categorical variables shown as *n* (%), continuous variables shown as mean (25th percentile, 75th percentile).

### Perioperative outcomes

Intraoperative data are described in Table 2. Mean bypass time for all techniques combined is 128 min, with a mean cross-clamp time of 94 min.

**Table 2:** Operative characteristics .

Variables	Patients, *n* = 28
Bypass time, min	128.3 [52–262] (54.8)
Crossclamp time, min	94.6 [29–168] (43.7)
Cardiac rhythm at CPB weaning	
Sinus rhythm	27 (96.4%)
Atrio ventricular Block	1 (3.6%)
Valvuloplasty technique	
Cusp plication	7 (25%)
Cusp resection	7 (25%)
Repair with a patch	14 (50%)
Direct suture on defect	0 (0%)
Bicuspidization	14 (50%)
Cusp reimplantation	3 (10.7%)
Not specified	1 (3.6%)
Annuloplasty	
None	17 (60.7%)
Sub coronary annuloplasty	5 (17.9%)
Double annuloplasty	6 (21.4%)
Associated procedures	
None	12 (42.9%)
Mitral valve plasty or replacement	3 (10.7%)
Septal myectomy	4 (14.3%)
Ascending aorta replacement	7 (25%)
Ventricular septal defect	2 (7.1%)
Ductus arteriosus closure	1 (3.6%)
Reclamping	3 (10.7%)

Categorical variables shown as *n* (%), continuous variables shown as mean (25th percentile, 75th percentile).

CPB, cardio-pulmonary bypass.

Sixteen patients (57.1%) patients underwent a combined procedure including ascending aortic replacement (25%), septal myectomy (14.3%), mitral valve surgery (10.7%) or ventricular septal defect surgery (7.1%) or patent ductus arteriosus closure (3.6%). Among aortic valve repair procedures, the use of a pericardial patch was necessary in the half of all patients. Bicuspidization was also frequently performed (50%), followed by cusp resection or plication (both 25%).

Eleven patients received external annuloplasty – five patients at the subcoronary level and six patients at the subcoronary and the JST. Five patients required aortic root remodelling (Yacoub technique); all of them had an associated subcoronary annuloplasty.

The only patient discharged from CPB with transitory atrioventricular block had undergone aortic annuloplasty.

There were 3 instances of a second aortic cross-clamp application (10.7%) in the cohort throughout the study, one for hemostasis, one for another surgical procedure on the mitral valve and missing data for the last patient.

Postoperative morbidity and mortality data are shown in the Table 3. The data do not differentiate between events occurring during the stay in intensive care or during hospitalization in conventional unit.

**Table 3:** Post operative data.

Variables	Patients, *n* = 28
Survival at discharge (transplant-free)	26 (92.85%)
Surgical site-infection	
Mediastinitis	1 (3.6%)
Endocarditis	0 (0%)
Stroke	0 (0%)
Bleeding requiring blood transfusion	4 (14.3%)
Pericardial effusion requiring drainage	2 (7.1%)
Atrioventricular block requiring pacemaker	0 (0%)
Myocardial infarction	1 (3.6%)
Need for ECMO	3 (10.7%)
ECMO time, days	4.7 [0–10] (2–7)
Phlebitis	1 (3.6%)
Acute renal failure	4 (14.3%)
Dialysis, days	1 (3.6%)
Length of stay in ICU, days	4 [1–29] (1–3)
Length of stay in hospital, days	13 [4–50] (8–11)
Mean weaning time from mechanical ventilation, days	1 [0–11] (0–0)
Amine withdrawal time, days	1 [0–17] (0–1)

Categorical variables shown as *n* (%), continuous variables shown as mean (25th percentile, 75th percentile).

ECMO, ExtraCorporeal Membrane Oxygenation; ICU, intensive care unit.

### Overall survival

Over the entire follow-up period, only one death in the cohort occurred during hospitalization (dissection of the ascending aorta and supra aortic vessels with tamponade, on the third postoperative day). Another patient required ECMO support due to inability to wean from CPB, in the setting of altered preoperative LV contractility; in the absence of recovery, he was put on the transplant list and was successfully transplanted and discharged from the hospital. Overall, survival at discharge (transplant-free) after the aortic valve repair was 92.85% (26/28 patients).

### Perioperative morbidity

The morbidity of aortic valve repair techniques was assessed using morbidity-producing events in the postoperative period. The most frequent events in the cohort were acute renal failure (14.3%), with 1 patient (3.6%) requiring renal replacement therapy, bleeding requiring transfusion (14.3%), and the need for pericardial drainage (7.1%). Three patients required ECMO cardiac assistance (10.7%) which lasted for a median of 4 days: one for dissection of the ascending aorta and supra-aortic trunks on the third post operative day (rescue ECMO); the second one for biventricular heart failure following failure to wean from the CPB, which was successfully weaned. The third one, with severe preoperative aortic regurgitation and left ventricular systolic dysfunction (LVEF 45%), also with failure to wean from CPB due to LV failure; he required orthotopic heart transplant after 7 days of mechanical support. Even though serial coronary angiograms and CT scans showed no impaired coronary perfusion while on ECMO, a morphological analysis of the explanted heart showed a high take-off of the left main coronary trunk, which put it at risk of external compression by the annuloplasty ring put in place at the JST, a complication which is not easy to foresee when the aorta is open and not under pulsatile systolic pressure.

The mean ICU stay was 2 days, and the mean total hospital stay was 13 days.

### TTE parameters evolution

All preoperative TTE were available, regarding TTE at discharge from hospital, one was missing (3.6%) - the patient who required mechanical circulatory support, and then rapid enrolment on the transplant list.

Concerning the last available ultrasound, we have missing data from three patients lost to follow-up and three patients who underwent reintervention (*n* = 20). The data collected on each TTE are shown in Table 4.

**Table 4:** TEE parameters evolution.

Variables	*N* = 28	Pre-operative	Hospital discharge	29 months post operative	*p*-value
LVEF,%	Mean [min-max] (sd)	69.7 [51–85] (10.4)	64.4 [45–96] (11)	68.7 [55–84] (8.5)	0.26
FS,%	Mean [min-max] (sd)	41.2 [27–67] (8.2)	38.6 [19.4–72] (11.4)	39.8 [27–54] (8.2)	0.80
LVEDD, mm	Mean [min-max] (sd)	48.8 [27–67] (12.2)	43.4 [27.2–60] (10.7)	44.7 [5–64] (13.8)	0.40
LVESD, mm	Mean [min-max] (sd)	29.2 [14–44] (9.4)	25.2 [9–44] (10.1)	30.2 [15–42] (7.2)	0.45
Mean AI grade *N* = 28		*N* = 28	*N* = 26	*N* = 20	
	Grade 0	3 (10.7%)	8 (30.7%)	3 (15%)	
	Grade 1	4 (14.3%)	10 (38.46%)	6 (30%)	
	Grade 2	5 (17.9%)	8 (30.7%)	8 (40%)	
	Grade 3	13 (46.4%)	0 (0%)	2 (10%)	
	Grade 4	3 (10.7%)	0 (0%)	1 (5%)	
Maximal aortic gradient, mmHg	Mean [min-max] (sd)	61.8 [4–130] (40.3)	31.3 [7–77] (17.8)	35.1 [0–104] (31.5)	0.02
Mean aortic gradient, mmHg	Mean [min-max] (sd)	37.3 [3–80] (24.3)	18.9 [8–39] (9.5)	18.2 [0–54] (19.2)	0.02
Aortic annulus diameter, mm	Mean [min-max] (sd)	20.5 [6,9–32.7] (7.1)	18.8 [10–32] (7.7)	21.2 [14–31] (5.5)	0.86

Categorical variables shown as *n* (%), continuous variables shown as mean (25th percentile, 75th percentile).

LVEF, left ventricular ejection fraction; FS, fractional shortening; LVEDD, left ventricle end-diastolic diameter; LVESD, left ventricular end-stage systole diameter; AI, aortic insufficiency; NA, not applicable.

Mean LVEF was 69.7% preoperatively vs. 64.4, 66.7% and 68.7% postoperatively, with no significant difference (*p* = 0.26). Left ventricular end-systolic and end-diastolic diameters showed no significant change over the entire follow-up period, with *p* values of 0.40 for LVEDD and 0.45 for LVESD respectively.

With regard to the evolution of the aortic ring diameter, the mean values were 21.5 mm, then 18.8 mm 20 mm and 21.5 mm. There was no statistically significant difference between these values (*p* = 0.86).

There was a significant difference in the evolution over time for maximal and mean aortic gradient with a *p*-value of 0.02.

Fisher tests do not show a more significant improvement in AI for repairs with patch vs. without patch during follow-up (Table 5). We have set the cut-off point of AI at grade 3 (moderate-severe), because this degree of regurgitation usually does not alleviate the preoperative condition of the patient; it requires a significant amount of medical treatment, regular monitoring by a cardiologist and carries an important risk of worsening symptoms, LV dilatation and surgical revision.

**Table 5:** Fisher test comparing aortic valvuloplasty with and without a patch during the hospitalization, and the follow-up (mean duration).

During hospitalization			9 months follow-up			29 months follow-up		
	Patch	Without patch		Patch	Without patch		Patch	Without patch
AI < 3	13	13	AI < 3	11	10	AI < 3	9	8
AI ≥ 3	0	0	AI ≥ 3	3	1	AI ≥ 3	2	1
*p*-value = 1			*p*-value = 0.6			*p*-value = 1		

AI, aortic insufficiency.

During the study period, 3 patients (11.53%) have required surgical revision of the aortic valve: 1 mechanical AVR and 2 Ross procedures.

**Figure 2:**
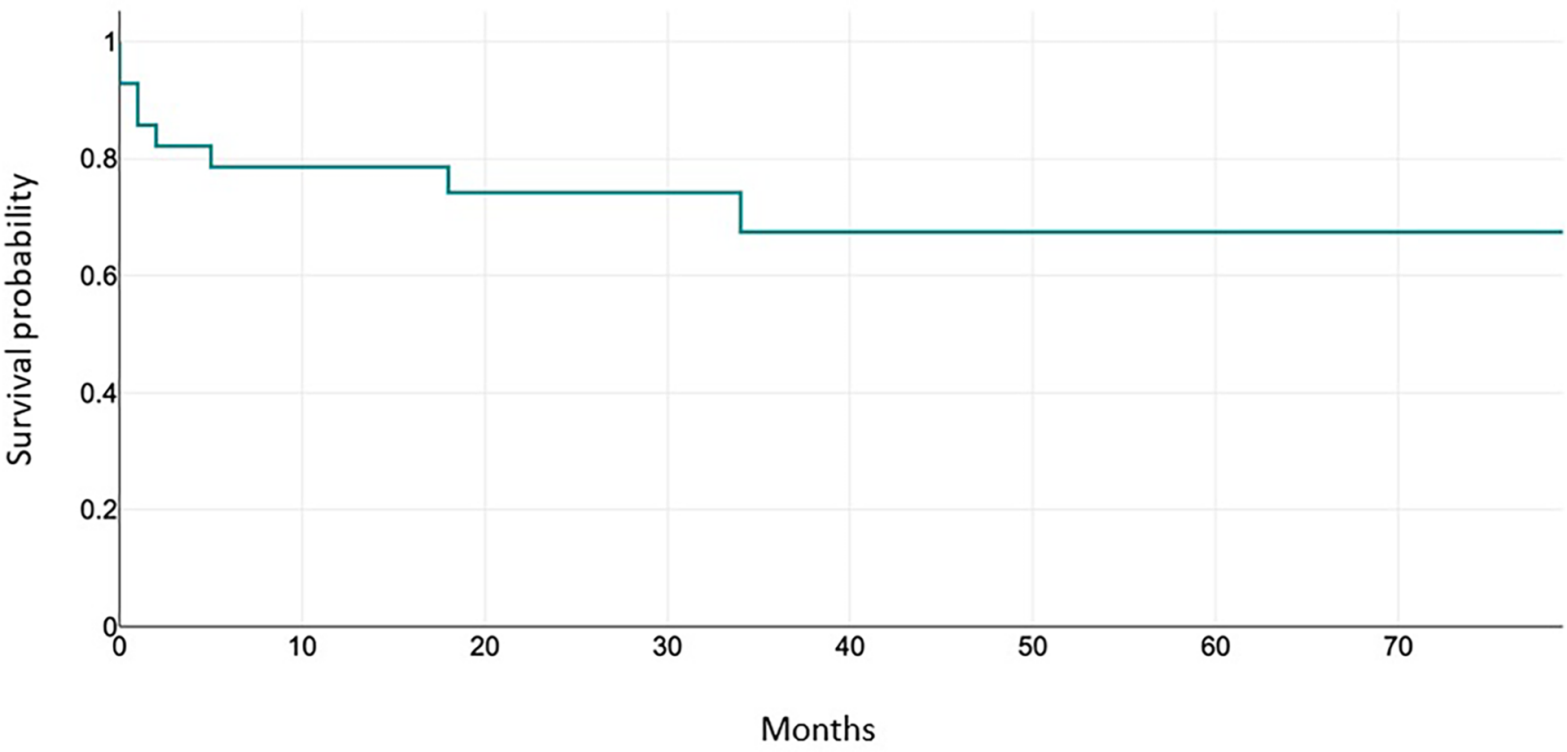
Freedom from valve-related reintervention over time.

Thus, the freedom from reintervention at 1 year is 89.7%, freedom from reintervention at 3 years is 85.7%. Results are shown in Figure 2.

## Discussion

### Key results

In this study, the cohort of patients operated on for complex aortic valve repair had an overall survival rate of 92.85% which is comparable to literature data (13, 14). Most were young adults with severe aortic insufficiency. Although there was no significant differences in aortic annulus diameter, it seems that these surgeries tend to improve IA grade or RA grade over time, at least in the short term, with a significative difference. The reoperation rate at 3 years is 14.3%, this value is also comparable to those found in the literature on the subject (13, 14). The morbidity associated with these techniques remains limited, since the mean length of stay for these patients is 2 days in the ICU and 13 days overall.

### Target population

The place of aortic valve repair remains unclear internationally (15). The European Society of Cardiology guidelines published in 2021 advocate aortic valve repair only in expert centers, in selected patients where lasting results are expected (16).

The choice of patients eligible for valve repair determines the success of the procedure. Valve morphology is a key factor, as is patient age, which is a good indicator of the risk of calcification. Previous studies have shown that valve retractions or tissue defects are not associated with good surgical results. Biscupid valves, on the other hand, were associated with excellent outcomes (17).

Patients with congenital heart disease are therefore the perfect target population for this type of technique, as they are young patients with a higher proportion of bicuspid valves than in the general population. Furthermore, the surgical teams who take on these patients are part of expert centers with a dedicated activity, which avoids the higher mortality risk associated with redo surgery (18).

### Surgical options

In the case of children, adolescents and young adults, the challenge of surgical reflection is to select the best option for these patients with their specific needs.

### Replacement

Aortic valve replacement raises a number of issues in the congenital population. The use of prosthetic valves exposes the patient to an increased risk of endocarditis, regardless of the type of prosthesis. In addition, the use of mechanical valves requires lifelong curative anticoagulation, with long-term compliance absolutely essential to avoid any further thrombo-embolic risk. Biological valves, used in young patients (particularly women of childbearing age), are subject to accelerated degeneration (19). Finally, the risk of mortality associated with the use of prosthetic valves is higher in younger patients (6).

The AVIATOR registry was set up to provide data on the long-term benefits of valve repair vs. valve replacement (20).

### Ross procedure

Aortic valve replacement using the Ross procedure is undoubtedly one of the best options for treating aortic root pathologies. It offers homograft growth potential, optimal hemodynamics and no need for long-term anticoagulation. Studies carried out on pediatric cohorts offer very encouraging results, with low mortality and reduced risk of infection and thromboembolism. The main obstacles using this technique are the secondary risk of dilatation of the homograft in the aortic position, transformation from a left-outlet pathology to an associated right-outlet pathology (21). Danial et al. compared the Ross procedure with aortic valve repair of complex lesions, and found similar outcomes in terms of survival (91% and 94.1% respectively), vs. 96.6% in our study. Concerning freedom from reintervention, their study found 69% for Ross technique, and 50.1% for aVR vs. 85.7% in our cohort at 3 years. They also found a trend towards less infectious endocarditis in group aVR (13).

### Ozaki technique

The Ozaki technique involves reconstructing the aortic valve with neo-cuspids of treated pericardium. As this is a recent procedure, long-term data are not yet available. However, population-based studies have been published with encouraging results (22).

### External annuloplasty

External annuloplasty has been strongly promoted as a means of stabilizing the aortic annulus, particularly in the case of bicuspid valves by reducing the size of the aortic annulus, thereby decreasing the risk of subsequent aortic insufficiency (23).

### Root surgery

In the case of aortic replacement, it would seem that aortic replacement with reimplantation technique appears to be a better strategy than remodelling in the congenital population, with a lower freedom from reintervention. In children or adults for whom aortic ring stabilization is envisaged, the results would appear to be comparable to aortic replacement (9).

### Aortic valve repair

Valve repair, for the reasons outlined above, remains an alternative that really needs to be considered in the therapeutic decision. The success of this procedure depends on several factors, not least the anatomy of the aortic valve, with the “anatomy-based repair concept” described for bicuspid valves (24). This makes it possible to detect certain lesions that are established risk factors for surgical repair failure such as asymmetrical bicuspid valve, defective cusps or calcifications. As a result, the use of pericardial patches has also been associated with an increased risk of failure (25). However, the CardioCel patch has the most advantageous characteristics for repairs in the aortic position compared with other heterologous pericardium patches (26), despite some early failures due to inflammatory intimal reactions and stenosis (27, 28). Further developments in biomaterial technology may address these limitations.

Another indicator of suboptimal outcomes is cusp retraction (type III disease). In the study by Boodhwani et al. from 2009 (29), patients with cusp restriction have a 5-year reoperation risk of 15%, compared to ∼5% in patients without cusp restriction.

As previously stated, it has become increasingly apparent that the reduction and stabilization of the functional aortic annulus is an important component of valve repair, especially in the case of bicuspid aortic valves. Adult patients with BAV and AI have significantly larger aortic annuli compared to patients with aortic stenosis and successful repair requires a 4–5 mm reduction in annular diameter (30). Although easily feasible in adults or adolescents, this reduction of annular diameter can be problematic in pediatric patients who have not yet finished their somatic growth. In these cases, where definitive reduction in diameter is not indicated, external annuloplasty can be forfeited, or alternative techniques (such as sub commissural plication) can be used. A relatively new internal annuloplasty ring has shown promising results in complex congenital patients, as reported by Lancaster et al. in 2023, with significant reduction in mean residual AI grade, no change in peak gradients, a freedom from reoperation of 97% at 2 years and a freedom from recurrent AI >=3 of 94% (31).

In contrast to mitral valve surgery, water tests are not entirely reliable for the aortic valve as a method to assess the quality of the repair. As a result, aVR failures are identifiable at declamping, and imply significantly increased bypass times and a second period of aortic cross-clamping in the event of a change of intraoperative strategy. It is therefore essential to select patients eligible for valve repair in advance, and to have a clearly defined strategy in case of failure.

### Limitations

Our study has several limitations due to its design: retrospective, monocentric, observational, short follow-up time, and a small number of patients. The results of our study should also be considered in the context of the learning curve inherent in any technique requiring expertise, and performed less frequently than other procedures (15, 19).

Some values are difficult to interpret, particularly those that change with age, such as the diameter of the aortic annulus, which should be studied by subgroup and with growth z-scores.

In conclusion, congenital aortic valve disease is a condition that patients have to live with for the rest of their lives. Recovery is not possible, and the medical and surgical management of these patients must be considered on a long-term perspective. The best treatment is the one with the fewest possible reoperations and the best associated quality of life.

## Data Availability

The raw data supporting the conclusions of this article will be made available by the authors, without undue reservation.
